# Pulmonary infection caused by *Tropheryma whipplei*: a case report and review of the literature

**DOI:** 10.1186/s13256-024-04936-y

**Published:** 2024-12-28

**Authors:** Jianglong Shi, Ren Liu, Jiehui Qiu, Chunping Wei, Dejin Pan, Tianxin Xiang, Na Cheng

**Affiliations:** 1 Jiangxi Medical Center for Critical Public Health Events, The First Affiliated Hospital of Nanchang University, Nanchang, 330052 Jiangxi People’s Republic of China; 2 Department of Infectious disease, The First Affiliated Hospital of Nanchang University, Nanchang, 330006 China; 3 Department of Hospital Infection Control, The First Affiliated Hospital of Nanchang University, Nanchang, 330006 China

**Keywords:** *Tropheryma whipplei*, Whipple’s disease, Pneumonia, Case report

## Abstract

**Background:**

*Tropheryma whipplei* pneumonia is an infrequent medical condition. The clinical symptoms associated with this disease are nonspecific, often resulting in misdiagnosis or missed diagnosis. Therefore, sharing and summarizing the experiences in the diagnosis and treatment of this disease can deepen global understanding and awareness of it.

**Case presentation:**

The patient is a 78-year-old married Han Chinese female who was admitted to the hospital after experiencing fever, dry cough, and fatigue for 4 days. A lung computed tomography scan revealed inflammatory exudation in the lower left lung, accompanied by pleural effusion. The bronchoalveolar lavage fluid was subjected to further analysis using metagenomic next-generation sequencing, which identified 41 genetic sequences associated with *Tropheryma whipplei*. Consequently, she was diagnosed with *Tropheryma whipplei* pneumonia. After initiating treatment with doxycycline and biapenem, the patient’s symptoms showed significant improvement. Upon discharge, the patient continued treatment with a combination of doxycycline and hydroxychloroquine, which was discontinued after 4 days. At 12-month follow-up, the patient reported overall good health, with no symptoms of fever, cough, or any other discomfort.

**Conclusion:**

*Tropheryma whipplei* pneumonia is a rare condition with nonspecific symptoms. The application of metagenomic next-generation sequencing technology in pulmonary infections helps to rapidly identify rare pathogens, providing a solid foundation for precise and effective antibacterial treatment for patients.

## Introduction

Whipple’s disease is a rare multisystem chronic condition with an incidence of less than 1 in 1,000,000 [1]. First described by George Whipple in 1907, the disease was initially believed to stem from a metabolic disorder owing to observed abnormalities in the digestive tract. However, with the advent of effective antibiotics and advancements in electron microscopy, it was later confirmed to be a bacterial infection. It was not until 2000 that the causative bacterium, *Tropheryma whipplei*, was successfully cultured [2, 3]. *Tropheryma whipplei* is a rod-shaped, Gram-positive bacterium, measuring approximately 2 µm in length and 0.25–0.5 µm in diameter. It possesses a distinctive three-layered cell wall ultrastructure, as observed under electron microscopy, and is recognized as one of the slowest-growing human pathogens, with a generation time of approximately 18 days [4]. Classic Whipple’s disease primarily affects the gastrointestinal tract, presenting with diarrhea and weight loss [3]. In recent years, cases of chronic localized and acute infections caused by this pathogen have been reported, including endocarditis, pulmonary infections, and ocular uveitis [5–7]. These new manifestations differ from the classical symptoms of Whipple’s disease and are more likely to lead to misdiagnosis or underdiagnosis. *Tropheryma whipplei* can cause either infection or colonization. In this case report, we describe a pulmonary infection caused by *Tropheryma whipplei* in an elderly female patient who presented with fever and a dry cough. Chest computed tomography (CT) revealed infectious lesions, but microbial cultures of sputum, blood, and bronchoalveolar lavage fluid (BALF) did not yield any identifiable pathogens. Fortunately, through next-generation metagenomic sequencing, we successfully identified *Tropheryma whipplei* in the BALF. Following targeted antimicrobial therapy, the patient’s condition improved, leading us to classify this as an infection rather than mere colonization. Furthermore, given the limited reports on *Tropheryma whipplei* pneumonia in both domestic and international literature, we conducted a retrospective analysis to enhance healthcare professionals’ understanding of this disease.

## Case presentation

**Fig. 1:**
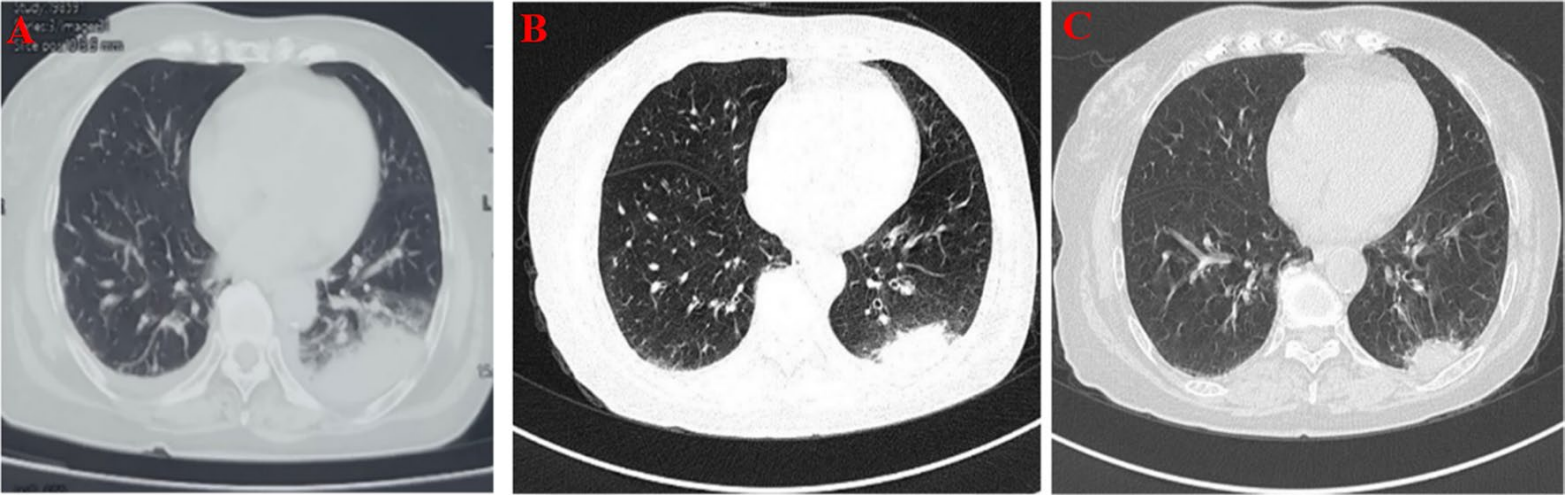
Chest computed tomography scan of the patient. The dates from left to right are 15 September 2021 (**A**), 23 September 2021 (**B**), and 29 September 2021 (**C**)

The patient is a 78-year-old married Han Chinese woman who was admitted to the hospital with a fever, dry cough, and fatigue lasting 4 days. She developed a fever 4 days prior, reaching a maximum temperature of 39.9 °C, accompanied by paroxysmal dry cough, fatigue, and thigh muscle pain, without any chest tightness or pain. On the third day of her illness, she visited a local hospital, where a computed tomography (CT) scan of the chest revealed inflammatory exudate in the left lower lung along with pleural effusion (Fig. 1A). Despite receiving empirical antiinfective treatment with moxifloxacin and oseltamivir phosphate, she continued to experience fever. During her illness, her mental state and appetite declined compared with her baseline health. The patient has a history of cerebral infarction and has been taking atorvastatin calcium tablets long-term. Additionally, she has a history of anxiety, for which she has been receiving ongoing treatment with haloperidol and melitracen. She also has a history of drug allergies, specifically to cephalosporins, penicillins, and azithromycin. There were no notable abnormalities reported in her personal, marital, or menstrual history, nor in her family history.

Upon admission, the patient’s body temperature was 37.8 °C, with a heart rate of 103 beats per minute and a respiratory rate of 20 breaths per minute. Her blood pressure was recorded at 136/68 mmHg (1 mmHg = 0.133 kPa). She appeared to be in poor mental state. Physical examination revealed a soft neck with no palpable enlargement of superficial lymph nodes. Auscultation of the lungs showed weak breath sounds bilaterally, but no significant rales were noted. The heart rate remained at 103 beats per minute, with a regular rhythm, and no murmurs were detected in any of the valve areas. The abdomen was soft, with no tenderness or pressure pain, and there was no edema in either lower extremity. Rebound tenderness was absent, bowel sounds were normal, and there was no swelling in the lower limbs. The patient was diagnosed with a pulmonary infection upon admission. Owing to the nonspecific nature of her symptoms, it is necessary to rule out other possibilities such as atypical pathogens, viral infections, and fungal infections when determining the causative agents of the pulmonary infection.

On the second day of hospitalization, during the ward round, we auscultated the lungs and noted the presence of some wet rales. Additionally, we conducted multiple laboratory and imaging analyses. The elevated levels of C-reactive protein (CRP) and erythrocyte sedimentation rate (ESR) indicated a significant inflammatory response in the patient. The specific results are as follows:

- **White blood cell count (WBC):** 4.99 × 10^9^/L (reference range: 3.5–9.5)
- **Hemoglobin (HB):** 93 g/L (130–175)
- **Platelets (PLT):** 143 × 10^9^/L (125–350)
- **C-reactive protein (CRP):** 105.41 mg/L (0–8)
- **Erythrocyte sedimentation rate (ESR):** 65 mm/hour (0–20)
- **Procalcitonin (PCT):** 0.15 ng/mL (< 0.5)
- **Interleukin-6 (IL-6):** 187.58 pg/mL (0–11.09)
- **Alanine aminotransferase (ALT):** 41 U/L (9–50)
- **Aspartate aminotransferase (AST):** 47 U/L (15–40)
- **Albumin (ALB):** 31 g/L (40–55)
- **Creatinine (CR):** 52.6 µmol/L (57–111)
- **Potassium (K):** 3.43 mmol/L (3.5–5.3)
- **Sodium (Na):** 125.6 mmol/L (137–147)

**Fig. 2:**
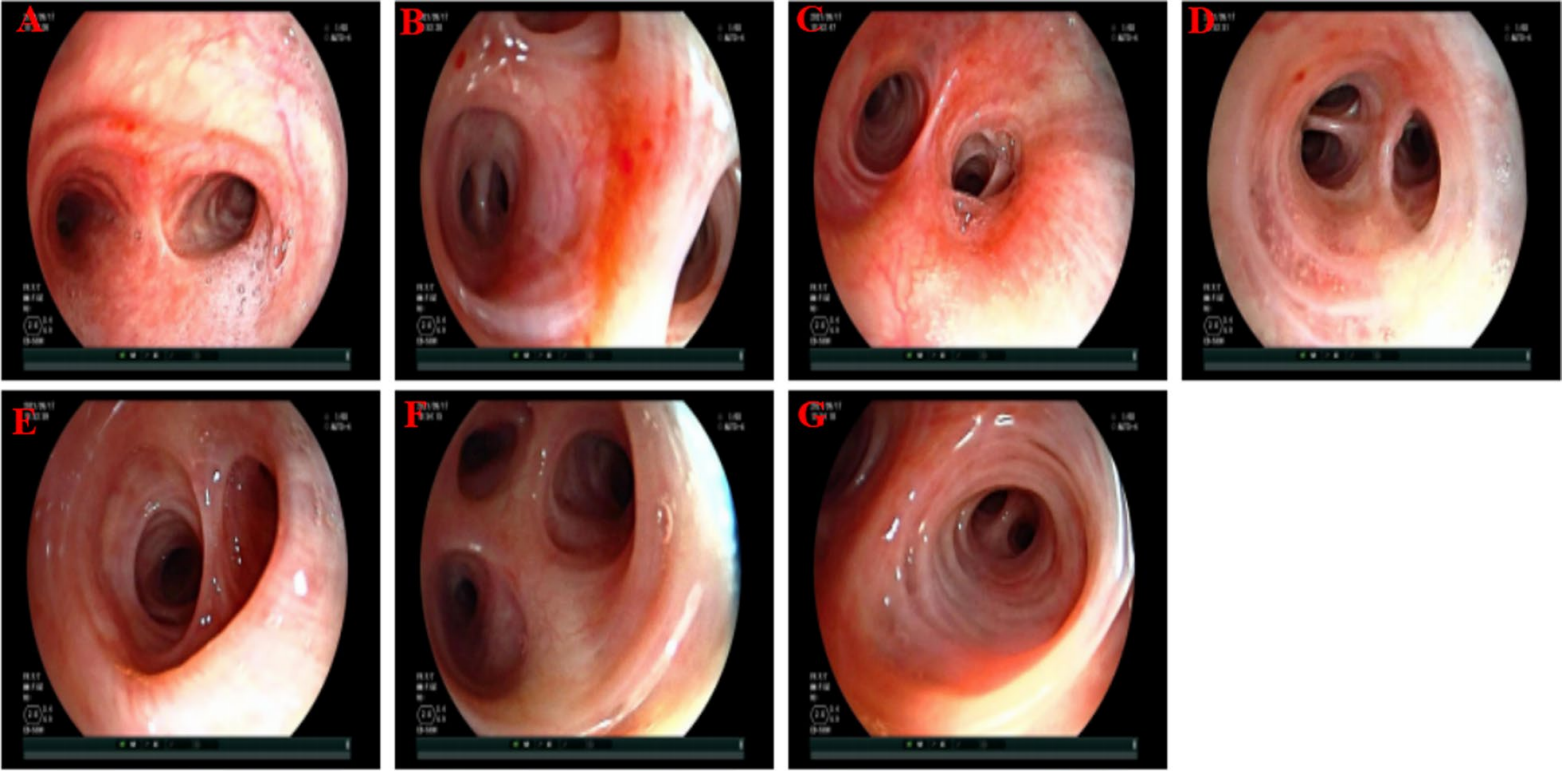
Fiberoptic bronchoscopy of the patient: trachea carina (**A**), superior lobe of right lung (**B**), middle segmental bronchus of right lung (**C**), middle lobe of right lung (**D**), lower lobe of right lung (**E**), inherent superior lobe of left lung (**F**), and lingual lobe of left lung (**G**)

*Mycoplasma pneumoniae* immunoglobulin M (IgM), *Chlamydia pneumoniae* IgM, respiratory syncytial virus IgM, adenovirus IgM, coxsackievirus group B IgM, plasma fungal-d-glucan, serum galactomannan, serum cryptococcal podococcal antigen, T-cell spot test for tuberculosis infection, tumor markers, urine and stool routine, coagulation function, electrocardiogram, and cardiac ultrasound were all normal. The thyroid ultrasound indicated bilateral lobar thyroid cysts, categorized as Chinese Thyroid Imaging Reports and Data Systems (C-TIRADS) 2. On the third day of hospitalization, bronchoscopy was performed, revealing a normal bronchial tree (Fig. 2). Subsequently, bronchoalveolar lavage was conducted, and the BAL fluid (BALF) was sent for testing using metagenomic next-generation sequencing (mNGS) at the Department of Laboratory, The First Affiliated Hospital of Nanchang University. On the fourth day of hospitalization, mNGS detected 41 sequence reads of *Tropheryma whipplei*. Additionally, no pathogens were found in the previously collected sputum and blood cultures. On the eighth day of hospitalization, the microbiological culture of the bronchoalveolar lavage fluid showed no pathogen growth. On the basis of her clinical symptoms, lung CT findings, negative microbiological cultures, and mNGS results, she was diagnosed with a pulmonary infection caused by *Tropheryma whipplei*.

Upon admission, the patient was treated with a combination therapy consisting of biapenem (0.6 g, intravenous, every 12 h) and doxycycline hydrochloride tablets (100 mg, oral, every 12 h), supplemented with supportive measures such as nutritional supplementation and maintenance of electrolyte balance. As a result, her temperature stabilized, and both her mental and physical strength significantly improved, with a substantial reduction in coughing. On the eighth day of hospitalization, laboratory evaluations revealed a CRP level of 14.65 mg/L, an ESR of 39 mm/h, a PCT of < 0.1 ng/mL, and an IL-6 level of 6.66 pg/mL. Liver and thyroid functions remained within normal limits, while chest CT scans demonstrated absorption of the infected lung lesions and a reduction in pleural effusion compared with previous scans (Fig. 1B). Given the slow resolution of lung inflammatory lesions, we conducted a thorough literature review on *Tropheryma whipplei* infections, considered alternative treatment options, and initiated hydroxychloroquine sulfate tablets (200 mg, oral, three times daily) on the 13th day. A follow-up chest CT on the 15th day showed further reduction in inflammatory lung lesions (Fig. 1C). Her temperature remained normal, coughing ceased, and her overall condition improved, leading to her discharge on the 16th day. Post discharge, she continued treatment with doxycycline hydrochloride tablets (100 mg, oral, every 12 h) and hydroxychloroquine sulfate tablets (200 mg, oral, three times daily) but discontinued self-administered treatment 4 days later. Her temperature remained normal without respiratory symptoms such as coughing or sputum production. The patient visited our outpatient clinic in late November 2021. A follow-up chest CT showed further reduction in pulmonary inflammatory lesions compared with 29 September 2021. In September 2022, during a telephone follow-up regarding her condition, the patient reported no discomfort and agreed to share her case publicly.

We utilized the PubMed database to conduct a comprehensive literature review, focusing exclusively on English-language papers. The search encompassed a timeframe stretching from the inception of the database until 28 November 2022, utilizing the search formula “[(Tropheryma Whipplei) OR (Whipple’s disease)] AND [(pneumonia) OR (Pulmonary infection)]”. Following the retrieval of articles, we meticulously reviewed the titles, abstracts, and full texts to exclude studies that were deemed irrelevant, retaining only those that unequivocally indicated pulmonary infection with *Tropheryma whipplei* for further scrutiny. Ultimately, eight studies met our criteria and were selected for analysis (Table 1). As depicted in Table 1, these eight studies encompass a cumulative total of nine case reports involving pulmonary infections caused by *Tropheryma whipplei*. Among these nine patients, six were male and three were female, with ages spanning from 23 to 81 years. All patients exhibited respiratory-related symptoms, including cough and dyspnea, while five presented with fever and four suffered from weight loss. Diagnosis was established through polymerase chain reaction (PCR) and next-generation sequencing (NGS) tests on bronchoalveolar lavage fluid or via histological biopsy. Post diagnosis, the majority of patients underwent a switch to sensitive antibiotics, such as tetracycline, doxycycline, hydroxychloroquine, and sulfamethoxazole, resulting in immediate symptom improvement. Upon monitoring their subsequent condition changes from the time of diagnosis, we observed that eight patients had a favorable prognosis, whereas only one had an unfavorable outcome. Upon reviewing the medical history of this latter patient, it was noted that he was of advanced age, had a prior history of coronary stenting and hypertension, was in a poor baseline condition, and NGS analysis of bronchoalveolar lavage fluid indicated a mixed infection involving *Tropheryma whipplei* and *Candida* sp. However, when his condition deteriorated, he declined extracorporeal membrane oxygenation, ultimately leading to his demise despite aggressive treatment efforts.

**Table 1:** Studies of pulmonary inflammation caused by *Tropheryma whipplei*

Case	Sex/age	Past medical history	Respiratory symptom	Other symptom	Diagnosis	Treatment	Follow-up* and outcome
Winberg CD [8]	M/52	Colonic polyp	Chronic cough	Fever, diarrhea, weight loss	Lung biopsy	Tetracycline	None
Kelly CA [9]	M/31	None	Dry cough	Weight loss, poor appetite	Histological examination	Penicillin, tetracycline	19 months, good
Fenollar F [10]	F/70	Gallbladder removal, scoliosis, diarrhea	Dyspnea	Nocturnal sweats, fever, myalgia,arthralgia	Lung biopsy and bronchoalveolar lavage fluid (PCR)	Doxycycline, hydroxychloroquine	8 months, good
Stein A [11]	M/24	AIDS	Dry cough, dyspnea	Fever, anorexia, asthenia, weight loss	Bronchoalveolar lavage fluid (PCR)	Cotrimoxazole	2 weeks, good
Wang S [12]	F/23	none	Chest distress, dyspnea	Fever, bloody stools and headache, joint pain, weight loss	Bronchoalveolar lavage fluid (NGS)	Imipenem	18 days, good
Yan J [13]	M/28	Pneumocystis pneumonia, AIDS	Dry cough, polypnea, chest tightness	None	Bronchoalveolar lavage fluid (NGS)	Sulfamethoxazole, meropenem	16 days, good
Zhu B [14]	M/44	Diabetes, hepatitis C, intravenous drug use	Dry cough, polypnea, chest tightness	None	Bronchoalveolar lavage fluid (NGS)	Trimethoprim, sulfamethoxazole	21 days, good
Li W [15], case 1	F/39	Cesarean section	Cough, dyspnea	Fever	Bronchoalveolar lavage fluid (NGS)	Sulfamethoxazole, meropenem	17 days, good
Li W [15], case 2	M/81	Coronary stenting and hypertension	Coughing, dyspnea	None	Bronchoalveolar lavage fluid (NGS)	Sulfamethoxazole, meropenem	2 days, poor

*M* male, *F* female, *NGS* next-generation sequencing, *PCR* polymerase chain reaction, *good* good prognosis, *poor* poor prognosis, *AIDS* acquired immunodeficiency syndrome

^*^The follow-up period for patients is counted from the time of diagnosis

## Discussion

Whipple’s disease is a multisystemic disorder, yet pulmonary involvement remains underreported. The cardinal clinical manifestations of lung involvement encompass cough, dyspnea, and chest pain, with CT scans revealing pulmonary nodules, interstitial alterations, and patchy infiltrates [16]. Owing to the absence of specific diagnostic markers, Whipple’s disease frequently goes unrecognized or misdiagnosed, often resulting in a delayed diagnosis. Prior to the advent of polymerase chain reaction (PCR) as a diagnostic tool, approximately 12 cases of classical Whipple’s disease were identified annually worldwide based on duodenal biopsy studies, although the actual incidence is likely much higher [17]. The current estimated global annual incidence ranges from 1 to 6 new cases per 10 million individuals [17]. Diagnosis of Whipple’s disease is challenging, with a median delay of over 6 years between the onset of initial symptoms and definitive diagnosis [18]. Currently, tissue biopsy, particularly with Periodic acid–Schiff (PAS) staining or immunohistochemical analysis of biopsy specimens, is widely regarded as the gold standard for diagnosing classical Whipple’s disease. In contrast, PCR testing of bodily fluids serves as the primary basis for diagnosing limited chronic infections. Microbiological cultures and serological tests are not routinely employed owing to their lack of specificity in blood tests [18]. In addition, in recent years, with advancements in microbial genetic testing technology, mNGS has played a significant role in diagnosing Whipple’s disease infections. mNGS is a novel pathogen detection technology that offers high efficiency, a broad spectrum of pathogens, and high sensitivity. It is particularly suitable for detecting rare pathogens. Compared with traditional methods, mNGS has demonstrated superior diagnostic performance [19]. However, mNGS also has notable shortcomings, such as its difficulty in distinguishing between pathogens and colonizing microorganisms. Additionally, the mNGS workflow is complex and involves multiple processes, which poses challenges to its widespread use. Apart from technical challenges, other factors limiting the broad implementation of mNGS include inadequate quality assurance, high costs, and considerations regarding sequencing depth [20]. *Tropheryma whipplei* infection can lead to classic Whipple’s disease, chronic localized infections, acute infections, and asymptomatic infections. The clinical symptoms of this disease are nonspecific, making it difficult for clinicians to detect. When patients present with frequent episodes of arthralgia, arthritis of large joints, seronegative polyarthritis, chronic diarrhea, malabsorption syndrome, abdominal pain, and unexplained weight loss, Whipple’s disease should be considered, prompting targeted investigations [21]. In terms of treatment, doxycycline combined with hydroxychloroquine constitutes an effective bactericidal regimen against *Tropheryma whipplei*, typically involving a 1-year course of doxycycline and hydroxychloroquine followed by lifelong doxycycline maintenance therapy. However, Whipple’s disease is characterized by a high recurrence rate, with 30–40% of patients experiencing relapses, necessitating regular monitoring. In the absence of clinical symptoms, biannual PCR monitoring of stool, saliva, and urine suffices [18, 22]. In reviewing the case we managed, we established a diagnosis of *Tropheryma whipplei* pneumonia based on the patient’s symptoms (fever with dry cough), imaging findings suggestive of pulmonary infection, negative microbiological culture results, and pathogenic genetic testing from bronchoalveolar lavage fluid. During the treatment process, we empirically initiated a regimen of doxycycline combined with biapenem upon admission, which led to rapid alleviation of the patient’s fatigue and dry cough, along with temperature control. This is consistent with the majority of reported diagnoses and treatment courses for Whipple’s disease.However, despite a 10-day course of antiinfective therapy, the patient’s lung CT showed minimal improvement in inflammation. At discharge, we adjusted the treatment plan to include doxycycline hydrochloride (100 mg, orally, every 12 h) and hydroxychloroquine sulfate (200 mg, orally, three times daily). Unfortunately, the patient did not adhere to the treatment regimen and discontinued her medication on her own 4 days post discharge. Despite the short duration of treatment, 1 year later, the patient reported no respiratory symptoms such as fever or cough, which contrasts with previous studies suggesting a treatment duration of over 1 year. Classic Whipple’s disease is a rare systemic condition typically characterized by weight loss, diarrhea, and arthralgia. Reports of acute pulmonary infections caused by *Tropheryma whipplei* are even rarer, and there are no large-scale clinical studies confirming the duration of therapy for Whipple’s pneumonia. Notably, the patient we managed did not exhibit symptoms of weight loss, diarrhea, or arthralgia; thus, we considered her condition to be an acute pulmonary infection caused by *Tropheryma whipplei*. Although she discontinued her medication 4 days after discharge, the absence of symptom recurrence suggests that the treatment duration for acute pulmonary infections caused by *Tropheryma whipplei* may not be as prolonged as previously thought. Further research with larger sample sizes is needed to validate the treatment duration for acute infections caused by *Tropheryma whipplei*.

## Conclusion

We report a rare case of pneumonia caused by *Tropheryma whipplei*. Whipple’s disease is a systemic infectious disease caused by *Tropheryma whipplei* that may affect multiple systems throughout the body. Whipple’s disease has a very low incidence and is difficult to diagnose. Metagenomic next-generation sequencing has good value in detecting *Tropheryma whipplei* infection.

## Data Availability

The medical data used in this study are available upon reasonable request to the corresponding author.
